# Effect of pre-session discrimination training on performance in a judgement bias test in dogs

**DOI:** 10.1007/s10071-024-01905-2

**Published:** 2024-10-12

**Authors:** Joseph Krahn, Amin Azadian, Camila Cavalli, Julia Miller, Alexandra Protopopova

**Affiliations:** 1 Animal Welfare Program, Faculty of Land and Food Systems, The University of British Columbia, Vancouver, Canada; 2 Department of Immunology, Pathophysiology and Veterinary Preventive Medicine, Wroclaw University of Environmental and Life Sciences, Wroclaw, Poland

**Keywords:** Cognition, Animal welfare, Canine, Affective state, Discrimination, Learning

## Abstract

Spatial judgement bias tests (JBTs) can involve teaching animals that a bowl provides a reward in one location but does not in another. The animal is then presented with the bowl placed between the rewarded and the unrewarded locations (i.e., ambiguous locations) and their latency to approach reflects expectation of reward or ‘optimism’. Some suggest that greater ‘optimism’ indicates better welfare. Performance in JBTs, however, may also indicate a learning history independently from welfare determinants. We hypothesized that dogs’ ‘optimism’ in a follow-up JBT may be impacted by a learning treatment involving additional trials of a different discrimination task. Once enrolled, companion dogs (*n* = 16) were required to complete three study phases: (1) a pre-treatment JBT, (2) a learning treatment, and (3) a post-treatment JBT. During the JBTs, dogs were presented with five locations: one rewarded, one unrewarded, and three ambiguous (all unrewarded). Dogs were randomly assigned to a trial-based learning task—a nose-touch to the palm of the hand. In the Experimental discrimination treatment phase (*n* = 8), dogs were presented with two hands in each trial and only rewarded for touching one specific hand. In the Control treatment phase (*n* = 8), dogs were presented with one hand per trial in alternating sequence and were yoked to dogs in the Experimental group to receive the same number of rewarded and unrewarded trials (to control for possible frustration). Using a repeated measures mixed model with JBT repeated within dog, we found no difference in the change in approach latency to the ambiguous locations between the dogs across treatments. ‘Optimism’ as measured in this JBT was not altered by the additional discrimination trials used in our study.

## Introduction

Determining the valence of an animal’s emotional state (i.e., affective state; how they *feel*) has value for understanding animals’ appraisal of their life conditions (reviewed in Fraser et al. 1997; Hemsworth et al. 2015) or interpretation of an experience (e.g., pharmaceutical intervention, Destrez et al. 2012; painful procedure, Neave et al. 2013). Understanding affective states can also be important for experimental control of individual variation (e.g., in biomedical research – see Bateson and Nettle 2015). However, affective state is necessarily subjective and cannot be directly measured (reviewed in Ede et al. 2019). Therefore, a variety of behavioural or cognitive tests have been developed to aid interpretation of non-human animal (hereafter “animal”) affective state, such as the judgement bias test (JBT), a frequently used paradigm to assess animal’s expectation of reward (‘optimism’).

Originally adapted for use in rats by Harding et al. (2004), JBTs are now widely used with procedures varying across and within species (for reviews see Gygax 2014; Roelofs et al. 2016). In a JBT, animals are taught to associate one stimulus (S^D^; e.g., location) with a reward and another stimulus (S^Δ^) with a less valuable reward, no reward, or punishment (S^ave^). They are then presented with ambiguous stimuli (e.g., a location between the S^D^ and S^Δ/ave^) and their behavioural response (e.g., latency to approach; approached or not approached) is used to assess the animal’s expectation of reward, which is operationally defined as ‘optimism’. In the case of latency as an outcome measure, slower approaches to the ambiguous stimuli are considered to represent more negative (‘pessimistic’) expectations while quicker approaches represent more positive (‘optimistic’) expectations (Harding et al. 2004; Mendl et al. 2009). A diverse array of cue modalities (such as auditory, spatial, olfactory, tactile, and visual cues), outcome measures (such as latency-based tasks, choice-based tasks), and task designs have been employed by previous research in the JBT (Hintze et al. 2018). Among these, spatial discrimination, which involves the ability to distinguish between locations, has been used in JBT designs across species (e.g., Hintze et al. 2018).

Outcomes of JBTs have been suggested to be influenced by both state differences within individuals and traits between individuals (reviewed in Roelofs et al. 2016, 2019). For instance, an animal’s ‘optimism’ in a JBT is often attributed to mood and affective state; within individual changes in JBTs are often used to demonstrate how individuals’ ‘optimism’ changes based on a potentially unpleasant situation (e.g., separation from mother, disbudding in cattle; Daros et al., 2014). It has also been suggested by some that greater ‘optimism’ in a JBT indicates greater welfare (e.g., Mendl et al. 2009; Vieira de Castro et al. 2020). However, some studies, have found no relationship between mood and the outcome of cognitive bias tests (e.g., Schick et al. 2013; Iigaya et al. 2016; discussed in Burani et al. 2020). Moreover, the outcomes of JBTs can be influenced by factors that may be independent from welfare determinants such as personality traits (dogs; Barnard et al. 2018), and sensitivity to reinforcers (rats; Rygula et al. 2012).

One area that has received recent attention is how learning/training influences animals’ behavioural responses in a JBT (e.g., see Mendl et al. 2009; reviewed in Nematipour et al. 2022).

As noted, the JBT involves an animal displaying a particular behaviour only when a specific stimulus is presented. This highlights the role of associative learning within the JBT, as being able to distinguish between stimuli is a vital component underlying performance on this test (Hall et al. 2021; Roelofs et al. 2019).

Discrimination is a fundamental way in which animals learn; a discriminative stimulus (S^D^) is an antecedent stimulus that signals to the animal that if they were to behave in a certain way, their behaviour would be reinforced. On the other hand, an extinction stimulus (S^Δ^) is an antecedent stimulus that signals no reinforcement for the target behaviour. Discrimination training has been adapted to many commonly used training procedures in dogs, including the training of working dogs (Hall et al. 2021) and reducing undesirable behaviours in companion dogs (e.g., Davidson and Rosales-Ruiz 2022). Dogs are frequently trained to distinguish between visuospatial cues in the context of agility and other sports (Gácsi et al. 2009; Marshall-Pescini et al. 2009). Other examples in which dogs engage in recognition and discrimination include finding a specific stimulus from a stimulus array of toys, objects, or locations (e.g., Dror et al. 2022).

Whereas discrimination learning is part of everyday life, researchers have also used formal discrimination training paradigms to ask animals various questions about their umwelt (e.g., Kelber et al. 2003). During a discrimination learning process, animals usually develop a response curve centered around the S^D^, generalizing their responses to comparable stimuli, including ambiguous stimuli that are more distant from the S^D^ (Strang and Muth 2023). In line with this, JBTs rely on animals’ differentiation of an S^D^ among similar stimuli, thus generating generalization gradients. The generalization gradient emerging in the JBT is based on the concept of associative learning such that stimuli which are more similar (e.g., closer to S^D^) will occasion more similar outcomes/responses (Blough 1969; Jamieson et al. 2012; reviewed in Roelofs et al. 2016). However, these generalization gradients that individuals express are not static and can be experimentally altered. For example, the introduction of an S^Δ/ave^ close to the S^D^ can result in a shift of the generalization gradient in the direction opposite to the S^Δ/ave^ (i.e., peak shift; Spence 1937; reviewed in Ghirlanda and Enquist 2003). Generalization gradients can also be influenced by an additional S^D^ (Blough 1969; reviewed in Ghirlanda and Enquist 2003). Moreover, the width of a generalization gradient can be narrowed with repeated exposure to a task or widened with increased duration between tasks (e.g., pigeons, Hoffman and Flescher, 1964; reviewed in Osnes and Lieblien, 2003). While some researchers have tested the effect of repeated JBTs on the resulting generalization gradients, the ambiguous stimuli in these experiments may lose their ambiguity as animals learn which stimuli are rewarded/unrewarded with repeated exposures (e.g., Wilson et al. 2023).

Other studies have tested how learning/training influence outcomes of a JBT in dogs. However, they often include potentially confounding effects as the training methods selected can influence both how an animal learns (expressed through their generalization gradient) and their affective state. For example, Duranton and Horowitz (2019) suggested that dogs trained in nose-work were more ‘optimistic’, reflecting better welfare (assuming that only the affective component of welfare was altered), compared to dogs trained for heeling exercises (i.e., teaching dogs to walk in a heel position). However, engaging in nose-work training may involve more training for stimulus discrimination (distinguishing between odours) compared to heeling. Other studies found that dogs were less ‘optimistic’ if trained using a greater proportion of aversive techniques (Vieira de Castro et al. 2020) or two or more aversive techniques (Casey et al. 2021). Aversive-based training (i.e., those using more frequent positive punishment/negative reinforcement methods) may affect mood (i.e., through repeated ‘unpleasant’ experiences), but perhaps also cause higher discrimination of voice/hand gestures than purely reward-based training (e.g., Hearst 1962). For example, aversive-based training can include the introduction of aversive consequences for responding under stimuli that are similar but not the same as the trained command (e.g., not moving from a stay position until a very precise release cue is provided and not when other similar utterances are made). Therefore, aversive training introduces a “cost” for the dog to engage in imprecise responses. In such cases, it is challenging to disentangle which factors are influencing the outcomes in the JBT.

Our study aimed to disentangle the effects of additional discrimination training from affective states in the results of a JBT in dogs, as we suspected that this additional training may alter the performance of dogs. Our objective was to determine if experiencing training focused on discrimination could affect the latency to engage with ambiguous stimuli during a JBT. To this end, dogs received an initial JBT, then underwent a discrimination training phase or a control phase, then received a second JBT. We hypothesized that there would be an interaction effect in which animals will behave more ‘pessimistically’ following discrimination training (i.e., longer latencies to approach ambiguous stimuli) compared to the control.

## Materials and methods

### Animals

This study was conducted between November 2022 and March 2023. Privately-owned companion dogs were recruited for this study from residents of Greater Vancouver, British Columbia, Canada. Dogs were required to be non-reactive towards humans, have received some form of nose-to-hand-touch training, and be able to come to the laboratory on three separate days for the three study phases. Once enrolled, all dogs were required to complete all three study phases: (1) a pre-treatment JBT (JBT1), (2) a hand-touch treatment phase, and (3) a post-treatment JBT (JBT2).

In total, 29 dogs were enrolled, but 13 were excluded for varying reasons: unable to meet the criteria of the acquisition phase in JBT1 (*n* = 8), dietary issues with rewards (*n* = 1), discontinued following weather-caused delays (*n* = 3), and researcher procedural error during the task (*n* = 1). We achieved a final sample size of *n* = 16; *n* = 8 in each treatment (Table 1). To the best of our ability, dogs in the treatment groups were balanced for sex (Discrimination, 5 male, 3 female; Control 4 male, 4 female), age (Discrimination, 3.2 ± 1.3 y, mean ± SD; Control, 4.5 ± 3.3 y), and breed clade (following Parker et al. 2017). Dogs varied in the intervals between JBT1 and treatment (5.8 ± 5.0 d; range: 1–22 d) and between treatment and JBT2 (5.7 ± 2.7 d; 1–10 d).

**Table 1:** The characteristics of the dogs used in the study. Numbering does not represent order of testing. Dog owner provided breed information and the clade was assessed following Parker et al. (2017). M, F, L, and R represent male, female, left, and right, respectively

Dog	Clade	Sex	Rewarded side	Age (years)	Treatment	Days between JBT 1 and treatment	Days between treatment and JBT2
1	UK rural	M	L	2	Discrimination	7	7
2	Mastiff	M	R	2.3	Discrimination	1	7
3	Pinscher	M	R	2	Discrimination	1	1
4	Pointer Setter	M	L	4.5	Discrimination	7	7
5	Drover	F	R	4.5	Discrimination	3	8
6	Terrier	M	L	5	Discrimination	7	1
7	Terrier	F	R	2	Discrimination	7	7
8	Mixed	F	L	2.9	Discrimination	4	10
9	UK rural	F	L	0.8	Control	3	7
10	Mixed	M	R	3.5	Control	5	7
11	UK rural	M	R	8	Control	8	4
12	Mixed	F	R	7.2	Control	3	7
13	UK rural	F	L	8	Control	1	4
14	Terrier	M	R	0.8	Control	7	1
15	Mixed	M	L	1.1	Control	22	7
16	Continental Herder	F	L	6.3	Control	7	6

Most dogs in the final sample (*n* = 13) were rewarded with chicken hotdogs (Maplelodge Farms^®^, Brampton, Ontario, Canada) and the reward size per trial was approximately proportional to dog bodyweight (~ 1 g hotdog/10kg dog). Owners supplied rewards for dogs with dietary restrictions (e.g., allergies; *n* = 3) and the reward size per trial was that which was normally given by the owner. Rewards were kept consistent in size and type within each individual across all trials and phases.

### General procedures

All tests involved three researchers hereafter referred to as “The Experimenter”, “The Timer”, and “The Handler”. During all study phases The Experimenter, The Timer, The Handler, and the dog’s owner wore mirrored sunglasses to limit the effect of inadvertent visual cues to the dog. Water was available *ad libitum* during all test procedures. Dogs were given approximately 5 min to habituate to the room prior to the beginning of tests in all of the phases.

### JBT general procedures

**Fig. 1:**
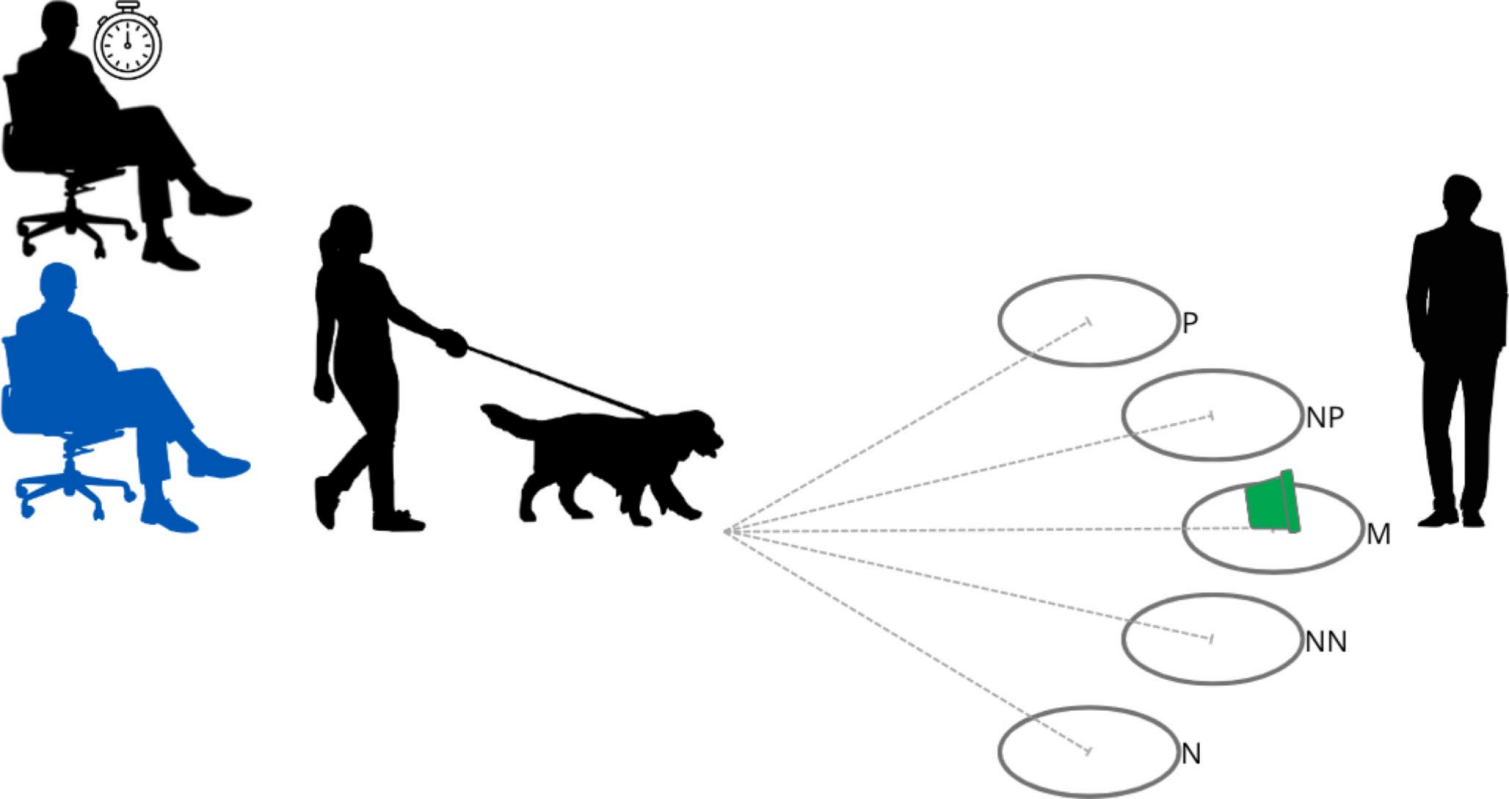
Before each experiment, five circles with a diameter of 1 m were drawn on the floor in chalk; the centre of each circle was 3.5 m from the starting position, represented by the dotted grey lines. These circles were 1.2 m and 20^O^ from the adjacent circle. At its centre, each circle had a 5 cm piece of adhesive hook and loop tape. The letters P, NP, M, NN, N indicating the location were drawn on the circle with chalk. The Timer was seated (next to the stopwatch, coloured black), the dog’s owner was seated (coloured blue), The Handler was next to the dog, and The Experimenter was standing behind the middle position

The JBT we used in our study followed the guidelines in Mendl et al. (2010a, b) with some modifications outlined in the brief description of each test below. The room had the same orientation for all dogs regardless of rewarded/positive (P) side (see Fig. 1). During both the acquisition and testing phase, The Timer had a pre-determined list of the order of locations where a bowl would be presented. There were five locations where the bowl was placed: positive (P), near-positive (NP), middle (M), near-negative (NN), and negative (N). Before each trial, The Timer would verbally indicate to The Experimenter the location to place the bowl (i.e., “positive”, “negative”, “NP”, “M”, “NN”). When located at position P, the bowl always contained a reward, while at N or the ambiguous locations (NP, M, NN) the bowl never contained a reward. When placing the bowl on the floor, The Experimenter would say “look” while looking at the bowl. The Experimenter would return to their central location and say “OK” to indicate to The Handler to release the dog and allow them to proceed to the bowl. The latency to engage with the bowl was measured from the time The Experimenter said “OK” to the time that the dogs’ first front paw crossed a 1 m diameter chalk circle surrounding the bowl. Similar to Duranton and Horowitz (2019), we used a maximum latency to approach the presented stimuli of 10s in all three phases (one dog waited for an additional 10s on two occasions, which was corrected to 10s for analysis). All latencies were measured with a stopwatch, with all times rounded to the nearest second. The orientation of the bowl presentation locations (i.e., P on left or right side of the room) was balanced across treatment groups and consistent within dogs across JBTs. To control for odour, we placed five treats between two non-air-tight containers that were used as the bowl in all trials. The bowl’s opening faced away from the dogs’ start position, limiting their ability to see the contents of the bowl until they crossed the chalk circle.

### JBT acquisition

All dogs were required to complete a pre-training (acquisition phase) before being tested in the JBT. In the acquisition phase dogs were presented with the bowl in one of two probe locations (N or P). All sessions began with two P then two N trials. Afterwards, each block of four trials following included two P and two N trials that were pseudo-randomly ordered such that there was never more than two of the same location presented consecutively. To complete the acquisition phase, dogs were required to have had a greater latency in the most recent three negative trials compared to the most recent three positive trials (Mendl et al. 2010a, b). Similar to Barnard et al. (2018), we set a maximum of 50 trials to meet the acquisition criteria. Dogs were required to complete a minimum of 15 trials (Mendl et al. 2010a). On one occasion, a dog met the criteria after 14 trials and the trial was terminated prematurely due to human error; this dog was included in all analyses.

### JBT testing

Latency in a JBT test was measured the same as in the pre-testing. In testing, all five locations were presented individually in the order as follows: M, NN, NP, NN, NP, M, NN, M, NP (Mendl et al. 2010a). This order was maintained for all tests to ensure each location was presented first, second, and third within a block of three trials (i.e., M, NN, NP), in an effort to minimize bias and improve comparability across subjects (as replicated from Wilson et al. 2023). Similar to Vieira de Castro (2020), each ambiguous location trial was separated from the next with one P and one N trial in random order.

### Treatment procedures

#### Treatment room set-up

**Fig. 2:**
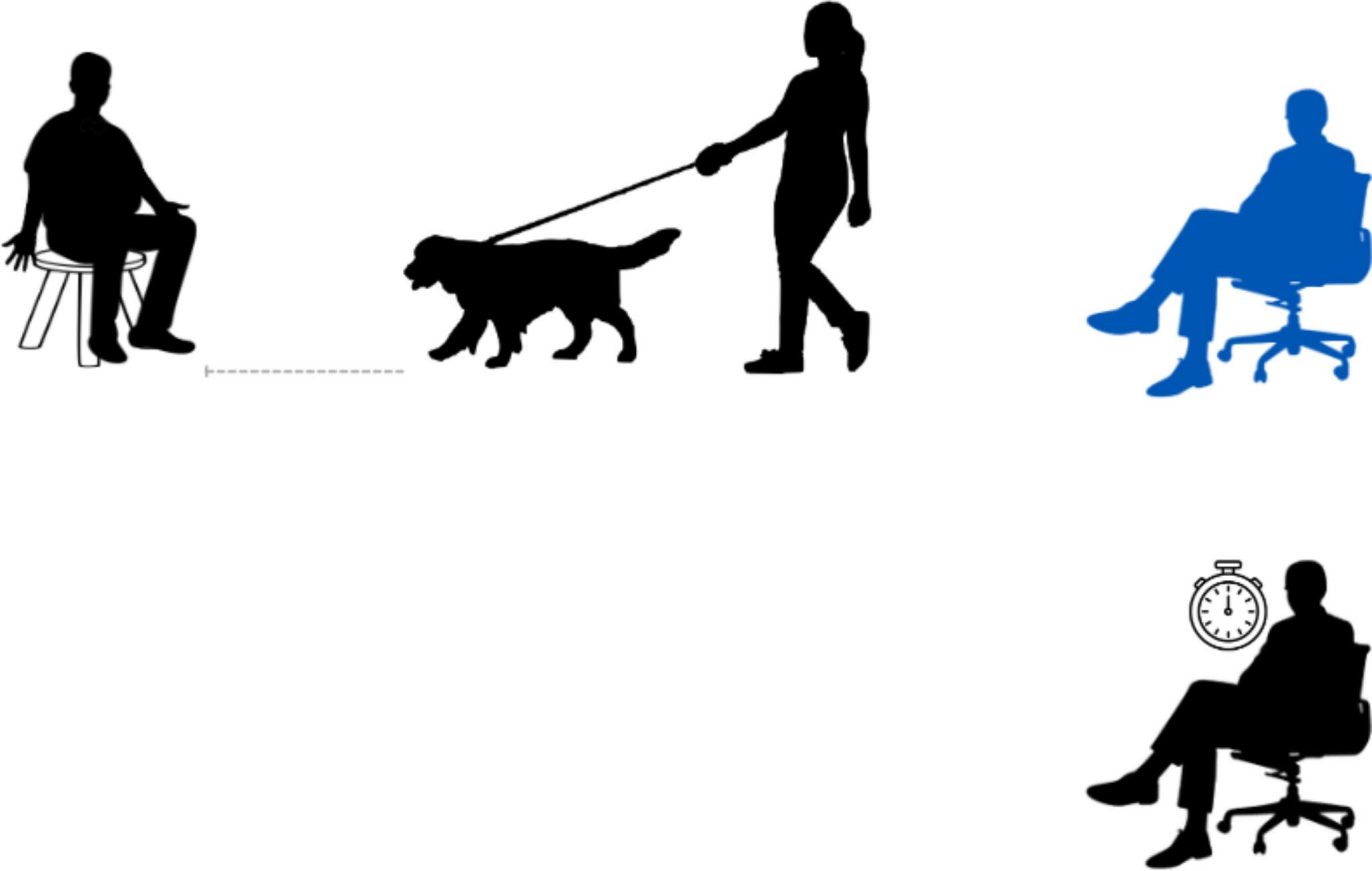
The Handler and the dog faced The Experimenter at a distance of 1.5 m, represented by the grey dotted line. The Timer was seated (next to the stopwatch, coloured black), the dog’s owner was seated (colored blue), The Handler walked the dog, and The Experimenter was seated in front of the dog

All procedures during the treatment phase had a consistent room layout (Fig. 2). The Experimenter was at the opposite side of the room than in the JBTs in an effort to minimize the similarity between the JBT and the treatment procedures.

#### Treatment acquisition

In the treatment acquisition, The Experimenter presented one hand at a time, alternating left and right. When The Experimenter would present the hand, The Handler would say “OK” and release any tension on the leash. When the dog touched the presented hand, The Experimenter would say “YES” and the dog received a reward. If a dog did not touch The Experimenter’s presented hand on one side five times in a row, they would be presented with the same hand twice. If they still did not touch the presented hand, a treat was placed between The Experimenter’s fingers of the presented hand so that it was visible to the dog (hereafter referred to as “Assistance”); the treat was provided when the hand was touched.

Dogs completed this acquisition phase once they consecutively touched four presented hands (two right, two left) without Assistance in ≤ 3s each. All dogs completed the acquisition phase with variation in the number of trials required (10.4 ± 11.4, mean ± SD).

### Discrimination treatment

In the Discrimination treatment (*n* = 8), the goal was to teach the dog that if they were to respond to one hand, but not the other, they would receive a reward. The Experimenter presented both hands simultaneously. The dog was only rewarded for touching one of the hands. In an effort to control for previous learning history across dogs with nose-to-hand touch in everyday life, the target hand was selected according to the owner’s non-dominant hand (left in all cases). If the dog did not touch either hand within 10s during two sequential presentations, they were presented with only the rewarded hand. If the presented hand was still not touched, they were provided with Assistance. After 10 presentations (i.e., one 10-trial block) dogs were given a 30s break. To complete this treatment phase, the dog was required to touch the rewarded hand 8 times in a 10-trial block. The 10-trial block was always completed except by one dog whose trial was prematurely terminated after 8 rewarded-hand touches (experimenter error). All dogs completed this stage requiring a varied number of trials (22.3 ± 10.2), resulting in a varied proportion of trials that were rewarded (70.0 ± 17.3%).

### Control treatment

The aim of the Control treatment was to establish a control condition where no hand is an S^D^. Dogs in the Control treatment (*n* = 8) were randomly match-paired to a dog in the Discrimination treatment to yoke the number of rewarded/unrewarded trials to be similar for dogs across trials. This was designed to control for potential differences in frustration, as non-rewarded trials are likely frustrating (e.g., Amsel and Ward 1965; Daly 1971), which may have resulted a negative emotional state (Amsel 1992). Because we aimed to disentangle the effects of learning and affective states, it was important to try to keep the dogs in a similar affective state (albeit this, of course, could not be fully controlled). Additionally, to ensure that no single hand resulted in a higher probability of rewarded trials, the number of unrewarded/rewarded trials was randomized such that there were no more than two unrewarded trials consecutively for both or either hand. In cases where there were an odd number of rewarded and unrewarded trials (*n* = 1), one side was rewarded once more than the other. Hands were presented one at a time in alternating sequence. If the hand that was pre-determined to be rewarded was not touched within 10s, Assistance was provided similarly to the Discrimination treatment.

### Data analysis and statistics

Analyses were conducted in R (version 4.0.3, R Foundation for Statistical Computing, Vienna, Austria) and SAS (version 9.04.01; SAS Institute Inc., Cary, NC, USA). We used a mixed model in SAS (Proc Mixed) with compound symmetry with JBT repeated within dog. The independent variable was the interaction between JBT (1 or 2) and the treatment (Discrimination or Control) and the dependent variable was the average change in latency to approach the ambiguous stimuli (combined NP, N, NN) during JBT1 and JBT2. We pooled the ambiguous locations as a simple approach to satisfy our model’s assumptions (and maintain the most appropriate degrees of freedom) because the approaches to the ambiguous stimuli were considered to be non-independent within a session. Other more complex nesting approaches for managing independence are possible in JBTs (see Gygax 2014 for an extended discussion). Due to factors outside of our control, the number of days between study phases (i.e., JBT1 to treatment and treatment to JBT2) varied across subjects. Therefore, we also included the interactions of JBT with the number of days from JBT to the treatment and JBT with the number of days between the treatment to JBT2. We conducted a second model, similar to the above model, but with the proportion of times the ambiguous locations were approached out of all trials at that location as the dependent variable (i.e., binary outcomes: approached or not approached within the 10s-time limit). The approach latency between NP and NN were average across all JBTs within individuals and compared using a student’s t-test in SAS (Proc Univariate).

## Results

**Fig. 3:**
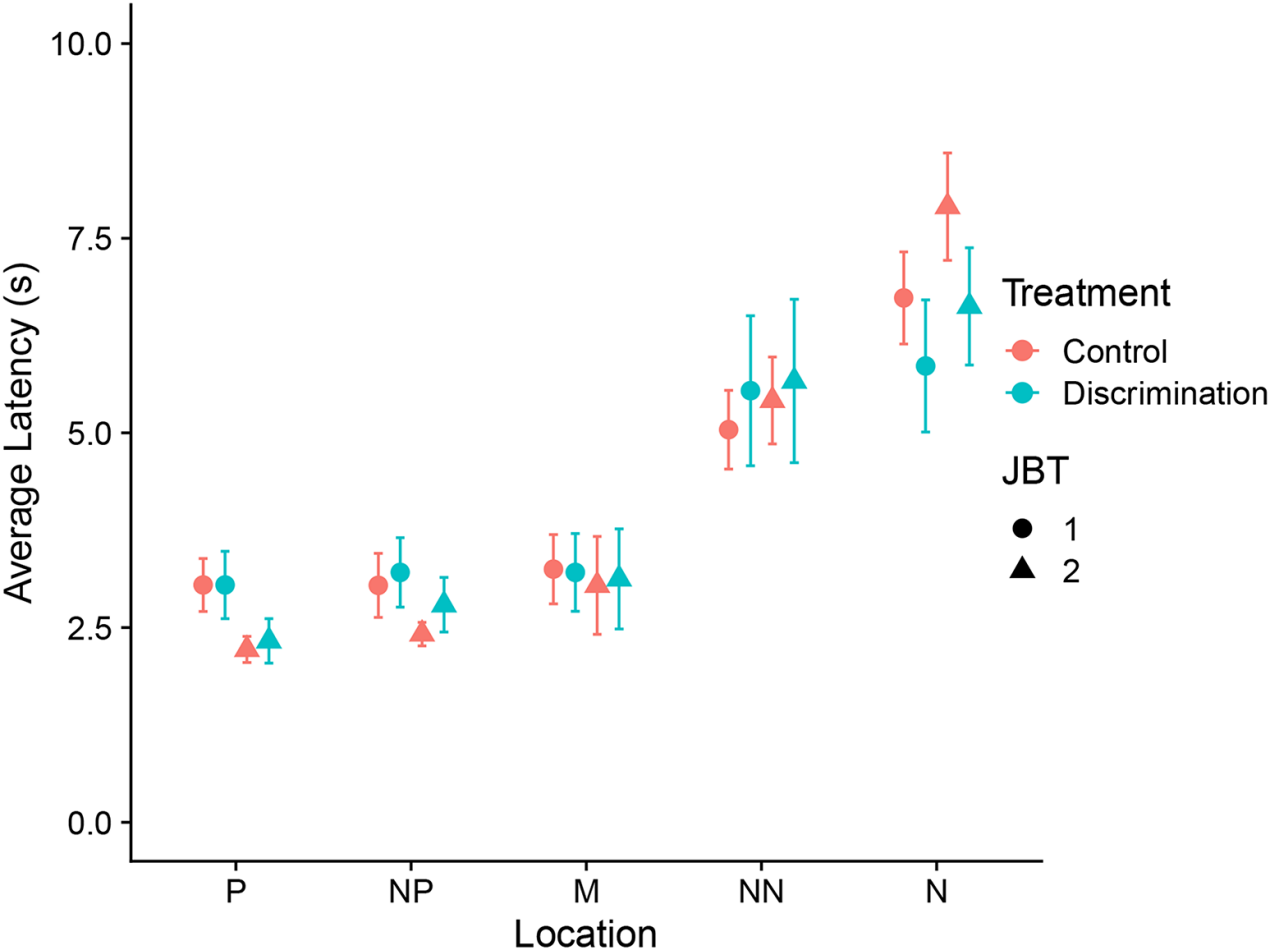
Dogs’ average latency to approach the five locations. Circles and triangles represent JBT1 and JBT2, respectively. Colour represents treatment. Error bars represent standard error of the mean

Of the 29 dogs that attempted JBT1, 8 (28%) failed to meet the acquisition criteria for JBT1. For the dogs that completed JBT1, the number of trials required to meet the acquisition criteria was similar between dogs in the two treatments (Discrimination: JBT1, 28.5 ± 5.1, mean ± SE; JBT2, 19.9 ± 2.5; Control: JBT1, 27.4 ± 3.6, JBT2, 18.0 ± 1.6). Probe location affected the dogs’ responses across JBTs with dogs having a greater latency to approach the near negative (5.4 ± 0.1) than near positive location (2.9 ± 0.2; t_15_ = 9.1, *P* < 0.001; Fig. 3).

**Fig. 4:**
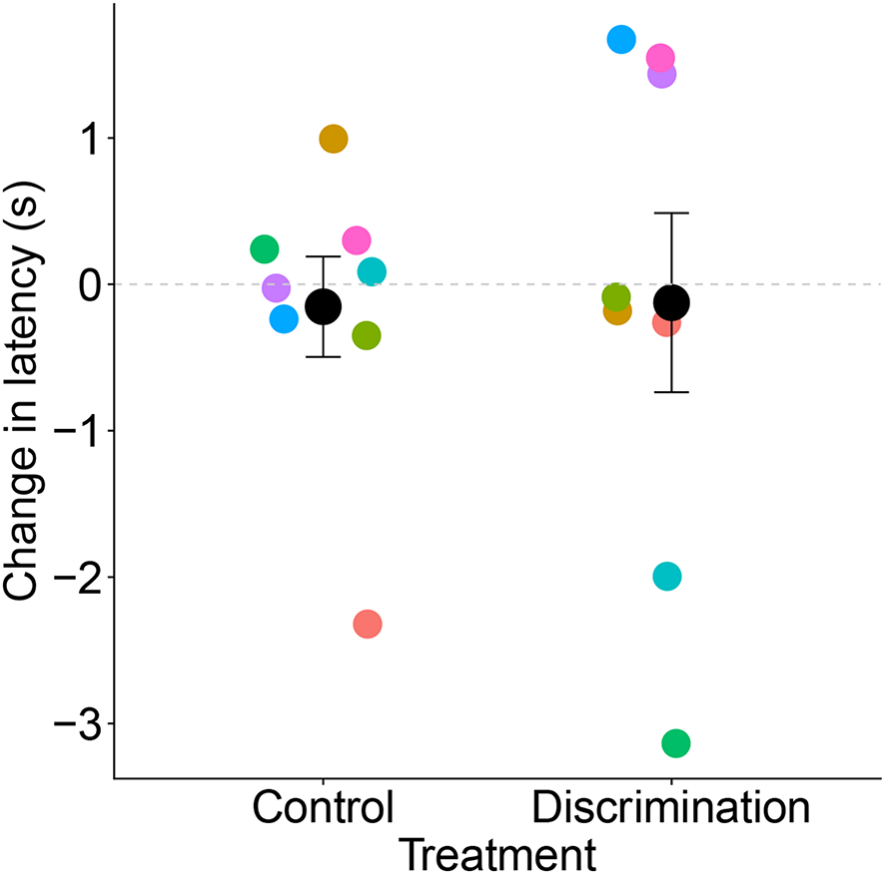
Points represent average change in the pooled latency to the ambiguous locations (NP, M, NN) from JBT1 to JBT2 per dog. Colours represent match-paired dogs. Black points represent means and error bars represent standard error of the means. The dashed line represents no change across JBT1 and JBT2

We found no difference in the change in approach latency to the ambiguous stimuli between the dogs in the Discrimination and Control treatments (F_3,9_ = 0.24, *P* = 0.86; Fig. 4). The number of days between study phases did not have a statistically significant effect on latency (JBT1 to treatment, F_2,9_ = 0.26, *P* = 0.78; treatment to JBT2, F_2,9_ = 2.00, *P* = 0.19). In the second model, the proportion of approaches to the ambiguous stimuli was also not affected by treatment (F_3,5_ = 0.13, *P* = 0.94) or the number of days between study phases (JBT1 to treatment, F_2,5_ = 0.39, *P* = 0.70; treatment to JBT2, F_2,9_ = 0.82, *P* = 0.49).

## Discussion

We set out to examine if a specific training experience focusing on visuospatial discrimination (where dogs were trained to discriminate between two visual stimuli (hands) based on their spatial orientation) but not involving stimuli similar to those involved in the JBT, could alter dogs’ responses to the ambiguous stimuli presented in a JBT. We hypothesized that discrimination training would narrow the stimulus response curve, thus increasing the response latency to ambiguous stimuli, therefore artificially creating the ‘pessimistic’ interpretation in the JBT. However, we found no effect of the training treatment on the dogs’ latency to approach the ambiguous locations in our JBTs. Our results may suggest that JBTs provide a robust method to investigate ‘optimism’ and affective state (e.g., mood)—an aspect of welfare—in dogs. Alternatively, responses in the JBT procedure may not be sensitive to the type of pre-session discrimination training used in this study, but could be generally sensitive to previous learning history (e.g., discussed in Bethell 2015; Wilson et al. 2023).

Importantly, our small sample size and the notable inter-individual variation (see Fig. 4) may have limited our ability to observe significant differences. Moreover, the treatment we used involved a different kind of stimulus (hand) compared to a JBT test (bowl), which may involve a different kind of spatial information for the dog, and a different procedure (e.g., dogs in the Discrimination treatment choosing between two response options in the same location versus approaching a single response option in different locations in the JBT). Perhaps our learning tasks were too dissimilar to the JBT procedure, thus limiting the priming effect of the learning task on the individual within the testing environment. Similarly, our treatment may have been too short in duration, did not have strict enough learning criteria, or had too long intervals between the treatment and retesting in JBT2 (e.g., the recency effect; Jones et al. 2006).

There were other limitations in our study design. For example, we rounded all times to the nearest second, reducing the precision (and potentially the accuracy) of our measurements. Similarly, we were unable to control for many aspects of the dogs’ lives as they were privately owned. For example, previous research found that stimulus features used in training can impact the degree of learning-dependent generalization (Wisniewski et al. 2009) and the stimulus response curve (Strang and Muth 2023). Therefore, the type of stimuli to which the dog was previously exposed could further influence performance on the JBT.

We set a maximum time of 10s in all procedures (similar to Duranton and Horowitz 2019). However, this meant that our latency measures were continuous until 10s at which point the outcome was treated as a test of whether the location was approached/not approached (similar to a go/no-go task, where dogs suppress a response to S^Δ^ rather than engage in a set behaviour procedure; e.g., see Daros et al., 2014). Although the proportion of trials where the dog approached/not approached was investigated as an additional outcome measure, there is still a possibility that this procedure may have affected the interpretation of our outcomes (see Gygax 2014).

While suggested by some as a gold standard for assessing animal emotions (e.g., Bateson and Nettle 2015), JBTs have faced scrutiny for a variety of factors, including low repeatability within individuals (e.g., Wilson et al. 2023) and variation across studies in methodology, contributing to variation in results (discussed in Roelofs et al. 2016; Lagisz et al. 2020; Lecorps et al. 2021). Similarly, statistical approaches in analyzing JBT data vary and are debated, lacking a unified approach (e.g., Gygax 2014; Bateson and Nettle 2015; Bethell 2015; Bethell and Koyama 2015; Burani et al. 2020). We treated the ambiguous locations as non-independent in our analysis (similar to Neave et al. 2013), although different statistical approaches may be more or less optimal in managing variability in the data and adhering to model assumptions.

Although some have considered that JBTs often have weak acquisition criteria (Roelofs et al. 2016), others have observed considerable failure rates where dogs do not meet the minimum acquisition criteria (e.g., 27% in Burani et al. 2020; 28% in this study) that biases studies to represent only those animals that are able to complete acquisition. Our use of a relatively neutral S^Δ^ rather than a punishment (S^ave^) at location N provided a smaller difference in pay-offs between locations N and P and possibly contributed to the considerable failure rates to meet the acquisition criteria (see Mendl et al. 2009). Similarly, there can be variation in the number of trials required to meet acquisition criteria that may not be included in assessing JBT outcomes (see Chan et al. 2023). We recommend future studies balance treatment groups by performance in JBT1 when possible; this variable was not included when balancing in our study. Moreover, the effect of hunger/satiation on measured outcomes (reviewed in Lecorps et al. 2021), and animals learning that the ambiguous cues are unrewarded (loss of ambiguity) within one JBT (Brilot et al. 2010) or across repeated JBTs (Doyle et al., 2010; in Roelofs et al. 2016) are common concerns that were not addressed in our experimental design. In some cases, methods have been developed to overcome these challenges. For instance, loss of ambiguity can be addressed by using partial reinforcement schedules at rewarded stimuli (e.g., Brilot et al. 2010; Neave et al. 2013) or a secondary reinforcer (e.g., clicker; Keen et al. 2014 in Roelofs et al. 2016). The sequence at which stimuli are presented can affect the interpretation of those stimuli (discussed in Jones et al. 2006; Zentall et al. 2014), a factor which was not completely controlled for in our experiment.

We encourage future research to continue critically assessing and refining measures of cognitive/judgement bias used to estimate affective state. For instance, studies could investigate further the effect of experimental procedures (interventions) designed to change this response gradient around the S^D^. This can be achieved, for example, by introducing stimuli outside of the S^D^ or S^Δ^ which may influence an animal’s outcomes in a JBT (i.e., peak shift; suggested by Gygax 2014; Roelofs et al. 2016). We also encourage continued discussion on statistical approaches in JBTs and that future studies investigate the implications of the considerable failure rates in some cognitive tests.

## Conclusions

We found no evidence that the learning treatment tasks affected ‘optimism’ (latency to approach an ambiguous stimulus) in dogs as measured using a JBT, providing no evidence against the robustness of the JBT in dogs. The considerable rate of dogs that failed to meet the acquisition criteria for the first JBT in our study may be a concern that we investigated only particular dogs who were, in some way, differently influenced by our procedures. We encourage future investigation into understanding how learning history can influence JBT outcomes, which will improve our understanding of the limitations of JBTs as a means to predict animals’ expectations and affective state.

## Data Availability

The datasets and analysis used in this study are available at https://doi.org/10.5683/SP3/QJLSKV.

## Declarations

### Researcher reflexivity statement

All authors have worked and/or lived closely with dogs for many years. This work was conducted in partial fulfillment of J.K.’s master’s degree. Publishing research papers has inherent value for scientists, which may be elevated for early-career scientists (see Rawat and Meena 2014). The analyses of data and framing of the research were inherently influenced by the values and interests of the authors and should be considered in the interpretation of this paper.
